# Cloning of the Repertoire of Individual *Plasmodium falciparum var* Genes Using Transformation Associated Recombination (TAR)

**DOI:** 10.1371/journal.pone.0017782

**Published:** 2011-03-07

**Authors:** Annette Gaida, Marion M. Becker, Christoph D. Schmid, Tobias Bühlmann, Edward J. Louis, Hans-Peter Beck

**Affiliations:** 1 Department for Medical Parasitology and Infection Biology, Swiss Tropical and Public Health Institute, Basel, Switzerland; 2 University of Basel, Basel, Switzerland; 3 Centre for Genetics and Genomics, The University of Nottingham, Queen's Medical Centre, Nottingham, United Kingdom; University of Montreal, Canada

## Abstract

One of the major virulence factors of the malaria causing parasite is the *Plasmodium falciparum* encoded erythrocyte membrane protein 1 (*Pf*EMP1). It is translocated to It the membrane of infected erythrocytes and expressed from approximately 60 *var* genes in a mutually exclusive manner. Switching of *var* genes allows the parasite to alter functional and antigenic properties of infected erythrocytes, to escape the immune defense and to establish chronic infections. We have developed an efficient method for isolating VAR genes from telomeric and other genome locations by adapting transformation-associated recombination (TAR) cloning, which can then be analyzed and sequenced. For this purpose, three plasmids each containing a homologous sequence representing the upstream regions of the group A, B, and C *var* genes and a sequence homologous to the conserved acidic terminal segment (ATS) of *var* genes were generated. Co-transfection with *P. falciparum* strain ITG2F6 genomic DNA in yeast cells yielded 200 TAR clones. The relative frequencies of clones from each group were not biased. Clones were screened by PCR, as well as Southern blotting, which revealed clones missed by PCR due to sequence mismatches with the primers. Selected clones were transformed into *E. coli* and further analyzed by RFLP and end sequencing. Physical analysis of 36 clones revealed 27 distinct types potentially representing 50% of the *var* gene repertoire. Three clones were selected for sequencing and assembled into single *var* gene containing contigs. This study demonstrates that it is possible to rapidly obtain the repertoire of *var* genes from *P. falciparum* within a single set of cloning experiments. This technique can be applied to individual isolates which will provide a detailed picture of the diversity of *var* genes in the field. This is a powerful tool to overcome the obstacles with cloning and assembly of multi-gene families by simultaneously cloning each member.

## Introduction

Malaria tropica is caused by the apicomplexan parasite *Plasmodium falciparum* and accounts for approximately 500 million cases with a death toll of 800'000 annually [1]. Nearly all pathology is caused by the intimate interaction between the infected erythrocyte and the host's capillary endothelium, which is conferred by a parasite-derived molecule in the membrane of the erythrocyte, termed erythrocyte membrane protein 1 (*Pf*EMP1). It is considered to be the major virulence factor of *P. falciparum* [2], [3]. *Pf*EMP1 shows antigenic variation and is encoded by about 60 different *var* genes per haploid genome. The expression of a *Pf*EMP1 variant is mutually exclusive [4], [5] and the protein is a major target of the adaptive immune response. It is believed that protection against clinical disease depends on the number of different *Pf*EMP1 variants previously seen by the immune system [6], [7]. A significant number of these proteins are able to bind to different endothelial receptors such as CD36 or ICAM-1, leading to sequestration of infected red blood cells from the blood stream, thus avoiding clearance by the immune system and the spleen [8]. Switching expression of *var* genes is often accompanied by differences in the binding phenotype, allowing the parasite to escape the host's immune response.

*var* genes vary in their size from 6 to 13 kb [9]. They are flanked by a large upstream promoter region that is considered to play an important role in the regulation of *var* genes [10], [11]. The *P. falciparum* sequencing project revealed the complete set of *var* genes of the 3D7 strain. They can be grouped into group A, B and C *var* genes according to their 5′ upstream regions [12], [13]. Group A and B *var* genes are located almost exclusively at telomere ends, where upsA *var* genes are transcribed towards the telomere and upsB *var* genes towards the centromere. In contrast, upsC *var* genes are located in central chromosomal regions [8]. Such distribution of group A, B, and C *var* genes has also been observed in parasite samples from naturally infected patients, however, sequence diversity appears to be vast, perhaps unlimited, with thousands of different Duffy-binding-like (DBL) domains already identified in parasite field isolates [14]–[16].

*var* genes possess a two exon structure with the first exon between 3.5 to 9 kb in size [12]. Exon 1 encodes the highly variable region that is expressed on the surface of infected red blood cells as well as a transmembrane domain. Each *var* gene comprises a distinct and unique set of domains which most probably have evolved through recombination from a common ancestor. The most conserved domain is the DBL1alpha domain with identities of up to 52% between different *var* genes [17]. Following an AT-rich intron, exon 2 encodes the more conserved amino terminal segment (ATS) that is located in the intracellular part of the infected red blood cell.

Sequencing downstream of the DBL1alpha domain poses difficulties because of the high AT richness, long homopolymeric sequence stretches, repeat units of various sizes and the fact that *var* genes evolve by frequent recombination. Both Sanger sequencing and next generation sequencing of whole genomes fail to unequivocally determine sequences of full-length *var* genes due to difficulties in the assembly of *var* genes *de novo* or onto existing scaffolds. To overcome these limitations we applied a technique that could specifically generate clones containing single *var* gene units. This information could provide important insights into the pathology of malaria. So far, only a very small number of *var* genes have been associated with distinct clinical presentations, such as the *var*2csa gene (PPL_003c) encoding a *Pf*EMP1 variant that binds to chondroitin sulfate A and is responsible for pregnancy-associated malaria [17]. Few studies were able to associate upstream regions of group A, B, and C *var* genes as well as the DBL1alpha domain in malaria infected children from endemic areas with clinical disease presentation [3], [15], [16], [18], [19], [20], [21]. However, analysis of complete *var* genes from patient samples might result in a different or more complete picture of parasite-host interactions involving *Pf*EMP1.

Transformation associated recombination (TAR) cloning has been successfully used for cloning of telomeric ends in various organisms [22], for cloning of centromere regions [23], [24], for gap closure of difficult sequence stretches [25], and for selectively cloning *vsg*–expression sites from *Trypanosoma brucei* [26], [27]. We applied a modified protocol of transformation-associated recombination [28], [29] to isolate individual *var* genes from *P. falciparum* strain ITG2F6. The Brazilian strain IT4/25/4, the parental strain of ITG2F6, has been partially sequenced and its *var* genes have been compared to those of 3D7 and HB3 [30]. This allows comparison of isolated *var* gene sequences of the ITG2F6 strain with IT4/25/4 sequences and facilitates sequence analysis.

TAR cloning offers several advantages over traditional cloning methods when genomic regions are difficult to deal with [29] as demonstrated with the cloning and characterization of bloodstream form expression sites from *T. brucei* [26], [27]. In the case of *var* genes, the alternative to TAR would be whole genome sequencing projects or the screening of random insert libraries rather than efficient targeted library construction using TAR. Here, we present the construction of a *var* clone library containing about 200 clones that were obtained in experiments using three different TAR cloning vectors specific for upsA, upsB and upsC *var* genes. We demonstrate the feasibility of analyzing these clones by PCR colony screening and Southern blotting. A selected number of *var* clones were transformed into *E. coli*, allowing plasmid preparations of high concentrations, for restriction fragment length polymorphism (RFLP) analyses or sequencing. Moreover, we used deep sequencing on three isolated *var* clones and were able to assemble them *de novo*.

TAR cloning can be used as a rapid and efficient method for the isolation of *var* genes of different *P. falciparum* strains. Moreover, utilization of this technique on field samples of infected patients will greatly support studies aiming to associate certain *var* gene types (present or expressed) with particular clinical presentations. Combined with long range PCR, this technique will provide a means to study the regulation and dynamics of *var* genes in naturally infected individuals and the associated functional significance of *var* gene expression that currently is an impossible task. Furthermore, it will provide data on the diversity of the entire full-length *var* gene repertoire.

## Results

### Construction of TAR library containing *var* genes from *P. falciparum* strain ITG2F6

**Figure 1:**
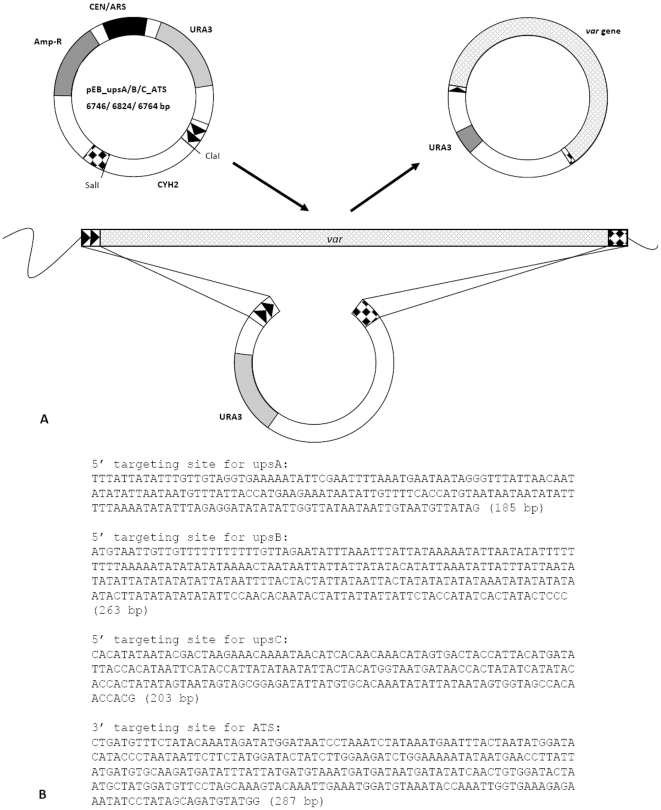
Construction of the *var* TAR vectors. (A) TAR cloning vectors pEB_upsA/B or C_ATS were constructed from the general TAR vector pEB2 (M. Becker and E.J. Louis, unpubl.). The 5′ targeting sites upsA (185 bp), upsB (263 bp) or upsC (203 bp) are indicated by black arrows. The 3′ targeting site is identical in all three TAR cloning vectors and contains part of the ATS region (287 bp) indicated with black squares. The yeast centromere and ARS are labelled as CEN and ARS, the positive selectable marker gene *URA3* is indicated as light grey box (URA3), the negative selectable marker *CYH2* with a white box (CYH2), and the Ampicillin resistance gene with a dark grey box (Amp-R). SalI and ClaI restriction sites are indicated. Homologous recombination between the targeting sites and *P. falciparum* genomic DNA results in the cloning of a *var* gene flanked by those sites as a circular molecule. (B) 5′ and 3′ targeting sequences used in the TAR cloning vectors pEB_upsA/B or C_ATS.

In order to isolate the repertoire of single *var* genes from the *P. falciparum* strain ITG2F6, a modified TAR cloning protocol was applied. Three TAR cloning vectors were designed to contain upstream sequences of group A, B and C *var* genes as 5′ targeting sites as well as the ATS domain as 3′ targeting site. In addition, they contained a yeast centromere, an autonomously replicating sequence (ARS), positive and negative selectable markers as well as an ori and *amp^R^* gene for replication in *E. coli* cells. Each of the three vectors was co-transformed with genomic DNA of the ITG2F6 strain into yeast spheroplasts. Homologous recombination within the spheroplasts resulted in circular yeast plasmids presumably containing a single *var* gene (Figure 1). We performed three independent experiments for each of the three TAR cloning vectors and obtained a total of 205 TAR clones of which 46 resulted from recombination with pEB_upsA_ATS, 91 from recombination with pEB_upsB_ATS, and 68 from recombination with pEB_upsC_ATS. This proportion is in accordance with the known distribution of *var* groups within the genomes of 3D7, IT4/25/4 and HB3 (Table S1).

### PCR screening and Southern blot analysis of TAR clones

**Figure 2:**
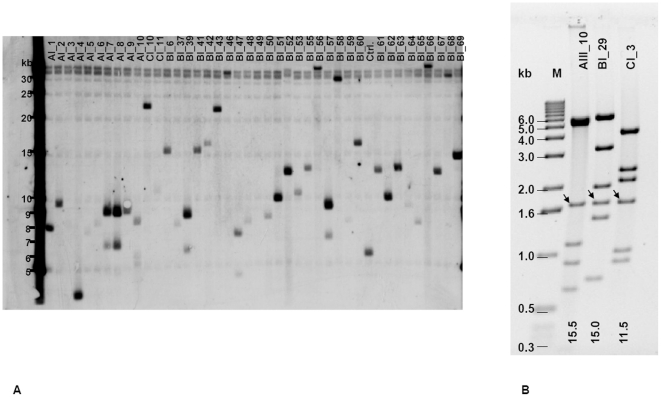
Southern blot analysis of *var* clones. (A) Southern blot example of 41 samples of NotI-digested genomic DNA of yeast TAR clones probed with vector-backbone. One lane contains a negative control (ctrl). (B) Restriction fragment pattern of TAR clones upsAIII_10, upsBI_29 and upsCI_3 digested with NotI and NdeI. The vector backbone yielded a band of 1600 bp and is marked with an arrow. The molecular marker is indicated. The upper band of the upsAIII_10 clones represents a double band.

In an initial PCR screen, using primers for the most conserved DBL1alpha domain, 48% of TAR clones were identified as positive, i.e. containing a *var* gene sequence. Subsequent Southern blot analysis conducted on a subset of 104 TAR clones identified 95 putative positive TAR clones (an example is shown in Figure 2), of which only 74 had been positive for the DBL1alpha domain by PCR. This demonstrates that DNA hybridization can reveal more positive clones than PCR due to variation in the primer region. Yeast colonies resulting from transformation with ‘empty’ vector without insert produced a band of approximately 6.0 kb (vector pEB upsA-ATS was used as negative control, Figure 2). We considered all clones larger than this as positive, taking into account the possibility that partial *var* genes could also be isolated. The additional 21 identified by Southern blotting may also contain *var* genes that are more divergent. A total of 119 putative TAR clones were identified with either or both methods within the 3 sets of experiments. TAR clone sizes ranged from less than 6 kb to approximately 30 kb (examples shown in Table 2). Sequencing of a 30 kb clone generated two contigs which could be linked by gap closure PCR yielding a total length of 11.5 kb which was further confirmed by RFLP analysis (see below).

### Restriction fragment Length polymorphism (RFLP) of selected TAR clones

In order to perform RFLP analysis, we transformed *E. coli* with DNA from yeast containing several candidate TAR clones. Despite the large size of the plasmids and the toxicity of yeast DNA to bacteria, we obtained 36 *E. coli* clones containing the TAR plasmids. To analyze the variability of the isolated TAR clones, we performed RFLP using restriction enzymes NotI and NdeI. The GC-rich NotI restriction site is unlikely to cut the AT-rich *P. falciparum var* gene sequence. There is a single NotI and one NdeI restriction site in the vector backbone of pEB2 which results in a distinct characteristic band of 1600 bp in all TAR clones. Screening the published 3D7 and IT4/4/25 *var* gene sequences, NdeI restriction sites were found to be present at one to eight locations in individual *var* genes, resulting in expected distinct banding patterns for different *var* gene inserts. All 36 *E. coli* clones were analyzed in this way. All upsA clones showed different NotI/NdeI digestion patterns indicating that different upsA *var* genes have been isolated. For upsB 23 TAR clones were analyzed, resulting in 20 unique patterns. One pattern was represented three times and one pattern was represented twice. The restriction digest of ten upsC clones revealed six patterns of which four were unique, one was represented four times and one twice.

These RFLP data demonstrate a high variability of isolated *var* genes. Although *var* gene IT4_var23p (Table 2) was represented four times, no bias towards cloning certain *var* genes was observed which could lead to the isolation of a restricted set of *var* genes.

Figure 2 shows the RFLP pattern of TAR clones upsAIII_10, upsBI_29 and upsCI_3, representative of all other examined clones. The total size of the bands summed to 15.5 kbp for upsAIII_10, 15 kbp for upsBI_29 and 11.5 kbp for upsCI_3. For upsAIII_10 and upsBI_29 this is consistent with the results obtained from Southern blot analysis and shotgun sequencing (Table 2 and section below), for clone upsCI_3 it is consistent with shotgun sequencing (section below).

### Sequence analysis of TAR clones

We sequenced a number of PCR-products obtained directly from the colony screening o TAR clones using both forward and reverse DBL1alpha -primers. As the degree of DBL1alpha conservation is only about 50% [17], annealing of the primers to different *var* genes is probably not optimal. The AT-richness of the *var* genes may explain why low sequence quality was obtained in some instances. However, BLAST analysis of all sequences confirmed the presence of *P. falciparum var* gene sequences, demonstrating that the TAR clones contained *var* gene inserts.

To confirm that nearly complete *var* genes were cloned, a representative clone for each of the different RFLP patterns was selected and 5′ and 3′ sequences were generated using primers located on either recombination site. Correct 5′ and 3′ *var* gene sequences were obtained for 30 clones. For two clones only a correct 3′ sequence was obtained. Three sequences represented group A, 20 sequences group B and nine group C *var* genes (Table 2). All sequences extended beyond the targeting sequence confirming successful recombination and cloning. As expected from the RFLP results, BLAST analysis gave identical hits for some TAR clones (Table 2). One clone, isolated with the upsC 5′ targeting sequence showed higher similarity to a group B *var* gene (PlasmoDB accession number PFD1015c) confirming previously observed limited fidelity within these two groups of *var* genes [16].

For each *var* group, we sequenced one yeast clone by deep sequencing (Illumina) and the sequences aligned well with the 5′ and 3′ sequences obtained by Sanger sequencing of the plasmids. For clone upsAIII_10 and clone upsBI_29 we were able to assemble all reads *de novo* into a single contig. We were able to identify the backbone of the TAR cloning vector pEB2, the 5′ targeting sites upsA and upsB, and the 3′ targeting site ATS in both contigs. BLAST searches were performed with the complete isolated *var* sequences against the NCBI non-redundant nucleotide database. Clone upsAIII_10 consisted of 11596 bp. BLAST search identified a partially sequenced *P. falciparum var* gene of the FCR3 strain (Accession number AJ133811) with 99% identity (13 mismatches over 9024 bp).

BLAST search of TAR clone upsBI_29 identified the IT4_var13 *var* gene (Accession number EF158072) with an identity of 99%. This similarity was expected since the strain ITG2F6 used here is a subclone of strain IT4/4/25. IT4_var13 is a group B *var* gene confirming the specificity of the upsB targeting sequence.

Sequencing of the upsC TAR clone upsCI_3 revealed two contigs each overlapping at one end with the pEB2 backbone. The gap between the contigs was closed by PCR using primers CI_3_pF and CI_3_pR (Table 1) corresponding to a sequence 90 bp before the unmatched end of either contig. The 500 bp fragment (Figure S1) resulted in an extremely AT rich sequence. The 5′ end generated with the forward primer showed a 100% match with the last 49 bp of contig 1 and similarly, the sequence generated with the reverse primer matched to the first 45 bp of the of contig 2 (Supplementary Figure 1). Further analysis revealed that the missing sequence stretch lies within the intron sequence. BLAST searches of the gap sequence resulted in a hit on the intron of *var* gene PFI0005w (Accession number AL844508). The complete sequence of clone upsCI_3 showed highest similarities with the partial IT4_var23 *var* gene (Accession number EF158077) belonging to group C *var* genes. This *var* gene has only been sequenced partially over 2049 bp displaying a 99% identity to the upsCI_3 TAR clone. The 3′ end of clone upsCI_3 showed an 85% identity with another partially sequence *var* gene (VARC1, Accession number AF100791).

All three isolated clones contained an open reading frame beginning with a methionine and were interrupted by an intron. The 3′ targeting sites of all three TAR cloning vectors do not cover the complete ATS domain, therefore the isolated *var* genes lack a stop codon in the second exon.

**Table 1:** Primers used for targeting, screening, sequencing, and gap closure.

primer name	sequence 5′to 3′
upsA_fw	tataatggatcctttattatatttgttgtaggtgaaa
upsA_rev	taatataagcttctataacattacaattattataacc
upsB_fw	tataatggatccatgtaattgttgtttttttttttgttag
upsB_rev	taatataagcttgggagtatagtgatatggtag
upsC_fw	tataatggatcccacatataatacgactaag
upsC_rev	taatataagcttcgtggttgtggctac
ATS_fw	tattatgtcgacctgatgtttctatacaaatag
ATS_rev	ataaatgtcgacgaattcccatacatctgctatagg
DBL1alpha _fw	gcaccgaagttttgcagatatwgg
DBL1alpha _rev	aartcttckgcccattcctcgaacca
CI_3_pF	ggatatatatatggatttacatg
CI_3_pR	gggtatatatatatatgtatgtg

Restriction sites are underlined. W denotes nucleotides A or T, R denotes A or G, and K denotes G or T.

## Discussion

We present the successful adaptation of the transformation-associated recombination (TAR) method [22] to isolate sets of individual *var* genes from the *P. falciparum* ITG2F6 strain. We obtained a library of 205 independent TAR clones, 98 (48%) of which tested positive for the presence of *var* gene sequence using the most conserved DBL1alpha domain in colony PCR screening. The low percentage of positives is a reflection of the limited conservation (around 50%) of the diagnostic DBL1alpha domain. PCR screening is adequate for first and fast results but is not reliable for the detection of the entire repertoire in the library. Using Southern blot analysis on a subset of 104 yeast clones 95 putative positives (91%) were detected, indicating that the library is likely to contain almost exclusively total or partial *var* genes. To further analyze these clones, 36 TAR clones were selected, transformed into *E. coli* cells and plasmids analyzed by restriction fragment length polymorphism (RFLP) and sequencing. As proof of principle, we further showed that pooling and bar-coding of three clones containing *P. falciparum var* genes allowed the subsequent *de novo* reassembly after deep sequencing (Illumina).

The use of yeast artificial chromosomes to clone large fragments of DNA dates back to the late 80s when Silverman and colleagues cloned segments of human chromosomes and specifically members of gene families [22], [31], [32]. Later, this technique was successfully applied to specifically clone human centromeric regions [23], [24]. TAR cloning was also used for gap closure in difficult areas in the human genome project [25]. With the development of higher sequencing capacity, it became feasible to target specific gene families by TAR cloning and an excellent example was the specific cloning of *T. brucei brucei*, *T.b. gambiense* and *T. equiperdum vsg* expression sites [26], [27].

The majority of *var* genes of *Plasmodium falciparum* are located in subtelomeric locations which represent approximately 10% of the whole genome. These areas are highly diverse and thought to undergo frequent recombination [33]. In addition, *var* genes are composed of repetitive domains with a high AT content. Sequencing projects struggle with the assembly of telomeric ends due to the limited information content in small sequence fragments from these areas. This poses large problems in the correct assembly of large multi-gene family genes such as *var* genes. Here we have shown that TAR cloning, which would generate clones containing only a single full-length *var* gene would greatly facilitate sequencing of the *var* gene repertoire of individual isolates overcoming the many ambiguities associated with such repetitive stretches.

To prove the feasibility and the principle of this approach we aimed at the specific isolation of *var* genes from the ITG2F6 parasite strain. We searched for conserved regions in the 5′ upstream regions which are used to define three groups and which show a relatively high similarity within but not between the groups [12], [13]. Based on the identified sequences we constructed for each of the groups a TAR cloning vector. We obtained 46 clones of group A *var* genes, 91 of group B *var* genes, and 68 clones of group C *var* genes and this distribution is broadly similar to the observed distribution of *var* genes in *P. falciparum* strains 3d7, It4, and HB3 [13], [30] (Supplemental Table 1). Sequence analysis of a number of obtained TAR clones demonstrated that most of the isolated *var* genes derived from the group which the cloning vectors had targeted, indicating that the selected targeting sequences occurred sufficiently specific to isolate *var* genes of a defined group. The one exception observed (one B clone obtained with the upsC vector) might be due to some alignment between sequences used as target sites for upsB and upsC *var* genes, which exhibit 44% identity.

Reliable screening of positive clones by PCR from colonies directly proved to be difficult. PCR amplification of a 400 bp fragment within the DBL1alpha domain, which is present in all known *var* genes (except in var2), gave a significant number of false negative clones (around 20%) based on subsequent Southern blot analysis. Although PCR tolerates some diversity, sequence conservation between DBL domains of as little as 52% [17] might be responsible for the missed sequences, particularly from colonies directly. In contrast, Southern blotting of total genomic or plasmid DNA identified many more potential positive clones. We considered all TAR clones with a size >6.5 kb as positive and demonstrated that all clones with total sizes above 9 kb contained *var* genes as confirmed by sequencing of the 5′ and 3′ ends of the insert.

RFLP analysis and sequencing of the 5′ and 3′ termini indicated that we isolated a broad range of different *var* genes. However, BLAST analysis using the sequence fragments and RFLP pattern also suggested some clones to be identical. The 5′ sequences of four TAR clones, i.e. upsCII_25, upsCI_3, upsCI_7 and upsCI_13 resulted in the same BLAST hit (Table 2) and three of those, upsCII_25, upsCI_3, upsCI_13, also revealed a similar RFLP pattern. The fourth clone, upsCI_7, had a different digestion pattern and thus was different in its overall sequence indicating that a large and diverse number of complete *var* genes were cloned.

**Table 2:** Comparison of selected TAR clones by Sanger sequencing of 5′ and 3′ ends of inserts and size analysis by Southern blot (a) and RFLP (b).

TAR clone	Blast result 5′sequence (gene-ID)	Blast result 3′ sequence (gene-ID)	*var* group	TAR clone size^a^	TAR clone size^b^
upsAI_2	IT4_var3	IT4_var3	upsA	9.5 kbp	8 kbp
upsAI_9	PFD0020c	VARC2	upsA	9.5 kbp	8.6 kbp
upsAIII_10	TM180var2	IT4_var54	upsA	15 kbp	15.5 kbp
upsBI_1	IT4_var54	IT4_var54	upsB	13.5 kbp	12 kbp
upsBI_2	MAL7P1.55	PFI0005w	upsBsh	13.5 kbp	11 kbp
upsBI_6	IT4_var41	IT4_var26	upsB	16 kbp	16 kbp
upsBI_8	Var13.1	IT4_var28	upsB	17 kbp	17 kbp
upsBI_11	Dd2_LT141	IT4_var13	upsB	16 kbp	15 kbp
upsBI_12	IT4_var26	IT4_var26	upsB	13 kbp	12 kbp
upsBI_14	IT4_var13	Dd2_LT141	upsB	16 kbp	17 kbp
upsBI_17	IT4_var11	IT4_var11	upsB	14 kbp	13.5 kbp
upsBI_21	var-3	var-3	upsB	15.5 kbp	14 kbp
upsBI_23	-	IT4_var24	?	10 kbp	11.5 kbp
upsBI_29	IT4_var13	Dd2_LT141	upsB	16.5 kbp	15 kbp
upsBI_31	var (EU787536)	IT4_var24	upsB	13.5 kbp	10.5 kbp
upsBI_32	PfEMP1(PFU31083)	FCR3-varT11-1	upsB	15 kbp	15 kbp
upsBI_33	var (EU787596)	IT4_var24	upsB	10 kbp	11 kbp
upsBI_35	var-3	PFI0005w	upsB	13 kbp	10 kbp
upsBI_38	IT4_var54	IT4_var54	upsB	13 kbp	15 kbp
upsBI_41	var-3	var-3	upsB	15.5 kbp	13 kbp
upsBI_61	var (AY462592)	IT4_var36	upsB	13 kbp	13 kbp
upsBII_1	IT4_var33	IT4_var33	upsB	n. d.	12 kbp
upsBII_3	var13.1	var13.1	upsB	n. d.	12.7 kbp
upsCI_3	IT4_var23p	PF07_0049	upsC	28 kbp	12.5 kbp
upsCI_10	Var (EU787639)	PFD0995c	upsC	23 kbp	10 kbp
upsCI_7	IT4_var23p	PFL1960w	upsC	14 kbp	12 kbp
upsCI_13	IT4_var23p	var13.1	upsC	>30 kbp	12.5 kbp
upsCII_6	PF07_0051	PFL1970w	upsC	n. d.	12.5 kbp
upsCII_14	PF07_0051	IT4var24	upsC	n. d.	13 kbp
upsCII_10	IT4_var40	PFD1015c	upsB	n. d.	13.5 kbp
upsCII_25	IT4_var23p	PF07_0049	upsC	n. d.	12.5 kbp
upsCIII_12	-	Varc2	?	13 kbp	12.5 kbp

*var* groups were determined by sequence alignment of targeting sites and from 5′ BLAST analysis. 5′ sequences of clones upsBI_23 and upsCIII_12 could not be determined consequently *var* group affiliation could not be determined. n.d. =  not done.

As proof principle, and to show that *de novo* assembly of *var* genes is possible with massively parallel sequencing, we analyzed three bar-coded TAR clones by Illumina sequencing. It is well known that AT richness and repeat structure of *var* genes poses large difficulties in the assembly of short sequence reads. However, we were able to assemble two out of three clones completely *de novo*. Clone upsBI_29 resulted in a single contig with 99% sequence identity to the coding sequence of the IT4var13 gene suggesting that the *de novo* assembled contig was correct. This gene previously has been isolated from the IT4/25/5 strain which is the parental strain of ITG2F6. Similarly, clone upsAIII_10 showed sequence identity of 99% to a *var* gene of the FCR3 strain associated with CSA binding. BLAST analysis found homologies in a stretch of approximately 1 kb in the 3′ sequence of a homologous incompletely sequenced *var* gene of the IT4/25/5 strain. Correct sequence assembly is confirmed by high sequence similarities to partially sequenced var genes derived from closely related strains. Using TAR cloning we were able to determine the full length sequence of these *var* genes. It is noteworthy that intron sequences in both TAR clones differed in length from the intron sequence of the annotated genes. This is likely to be due to the extremely high AT richness of the introns. The third clone, upsCI_3, comprised two contigs which we were able to link by PCR and Sanger sequencing. The inconsistency between the size of this clone determined by Southern analysis and the RFLP and sequence analysis could be due to a variety of issues such as a plasmid dimer in yeast. The PCR product spanning the gap was corresponded to the intron and was as such extremely AT-rich. Therefore, we could not fully determine the length of the gap sequence, highlighting the difficulties to assemble such DNA domains in the genome sequencing projects.

In conclusion, we have shown that TAR cloning can be used to specifically clone members of gene families in *P. falciparum* and this technology can now be applied to field samples to study the full diversity of *var* genes. It might be possible to clone full length *var* genes from cDNA which would allow association of expressed *var* genes with distinct phenotypes. This could provide enormous insight into the presence of phenotype-associated *var* genes. Having the predicted amino acid sequence of such genes could shed light onto the true associations with disease, as full length *Pf*EMP1 molecules might confer different binding affinities than cloned short fragments out of the protein context. TAR cloning could become a major tool for functional elucidation of multigene families in *P. falciparum*. It also could be applied to specifically isolate members of other multigene-families within the *P. falciparum* genome, such as the *repetitive interspersed family* (*rif*) or the *subtelomeric open reading frame* (*stevor*) genes [34]-[36]. Similar to *var* genes, these genes often cluster at subtelomeric regions. TAR cloning could be applied to isolate telomere ends and larger chromosomal regions and thus increase our knowledge on these important regions in *P. falciparum*.

## Methods

### *P. falciparum* strain

Parasites of *P. falciparum* strain ITG2F6 were cultured at 5% hematocrit as previously described [37], using RPMI medium supplemented with 0.5% Albumax [38]. Cells were lysed in 0.03% saponin in PBS for 10 minutes on ice, centrifuged at 4000 x g for 10 minutes and washed in PBS until the supernatant was clear. The parasite containing pellet was subjected to proteinase K digestion and genomic DNA was subsequently prepared by phenol/chloroform precipitation [39].

### *S. cerevisiae* strain

The *S. cerevisiae* strain TYC1 (genotype: MATa, ura3-52, leu2Δ1, cyh2^r^, containing a knockout of DNA Ligase 4) was used as the yeast host in TAR experiments as previously published [26].

### Construction of *var* TAR cloning vectors

*var* TAR cloning vectors pEB_upsA-ATS, pEB_upsB-ATS and pEB_upsC-ATS, respectively were constructed from the basic TAR cloning vector pEB2 (M M Becker and E J Louis, unpublished). They contain the yeast selectable marker *URA3*, the negative-selection marker CYH2 [40], a yeast centromere and an autonomously replicating sequence (ARS). In addition the vector contains an origin of replication and the *amp^R^* gene for growth and selection in *E. coli* (Figure 1)

For the use as 5′ targeting sites, homologous regions of the 5′ untranslated regions of group A, B, and C *var* genes were individually cloned into vector pEB2 upstream of the *CYH2* gene. A 292 bp fragment homologous to the acidic terminal sequence (ATS) of *var* genes was cloned into all three vectors downstream of the *CYH2* gene as 3′ targeting site (Figure 1). The 5′ homologous regions were 185 bp long for the group A genes, and 263 and 203 bp for the group B and C *var* genes, respectively. All 5′ targeting sites were chosen based on alignments of partially sequenced field samples (Accession numbers EU787517.1 – EU787984.1, AY462581.1 – AY462851.1) and the published 3d7 *var* gene sequences. For the 3′ targeting site only 3d7 *var* gene sequences were aligned, as no sequence information of field samples was available. The most conserved sequence stretches were chosen as 5′ and 3′ targeting sites, and were amplified by PCR from ITG2F6 genomic DNA using the primers shown in Table 1. Initially, all amplified targeting sequences were cloned into a pGEXT-vector and several clones per group were sequenced. For each target site, the sequence that occurred with the highest frequency and highest similarity to the original consensus sequence, was moved into pEB2, to form vector constructs as shown in Figure 1. The 5′targeting sites for group A, group B, and group C *var* genes were cloned using restriction sites BamHI and HindIII into pEB2. The 3′targeting site containing a sequence stretch homologous to the ATS domain was cloned via SalI/XhoI into pEB2. Clones were sequenced again to confirm the correct orientation of the targeting sites. Prior to transformation into yeast spheroplasts all vectors were digested with ClaI and SalI restriction enzymes to linearize the plasmids and to release the *CYH2* gene.

### TAR procedure

The TAR cloning procedure was essentially performed according to Kouprina and Larionov [29] with the following modifications: Yeast cells were incubated with zymolyase solution (10 µg/ml) for 30 min at 30°C, for each transformation reaction 450 µl of yeast spheroplast solution was used, 4.5ml PEG 8000 solution was used and spheroplasts were resuspended in 1ml SOS solution. Spheroplasts from each transformation reaction were mixed with 15 ml melted synthetic complete agarose medium lacking uracil but containing 1 M Sorbitol and 10 µg/ml cycloheximide and poured onto plates containing the same medium. This allows for positive selection of the TAR vector that provides *URA3* function. The addition of cycloheximide prevents transformation of empty plasmids, which remain circular due to incomplete ClaI and SalI restriction digest, as those vectors still maintain the *CYH2* gene that confers dominant sensitivity to cycloheximide. A full manual can be downloaded from www.nottingham.ac.uk/biology/people/louis/principleoftarcloning.

### Identification of *var* gene positive TAR transformants

TAR transformants, grown selective medium, were initially screened by colony PCR using primers for the DBL1alpha domain (Table 1). Single yeast colonies were suspended in a mix of 10 µM pF_DBL1alpha, 10 µM pR_DBL1alpha (Table 1), 0.4 µl Taq and 0.9 µl 11.1xPCR-buffer (45 mM Tris-HCl (pH 8.8), 11 mM ammoniumsulphate, 4.5 mM MgCl_2_, 6.7 mM 2-mercaptoethanol, 4.4 mM EDTA (pH 8.0), 113 µg/ml BSA, 1 mM dATP, dTTP, dCTP and dGTP) and adjusted with H_2_O to a final volume of 10 µl. PCR conditions were as follows: 5 min at 95°C, 40 cycles of 30 sec 94°C, 40 sec 57°C and 2 min 72°C and a final step of 5 min at 75°C.

All yeast TAR clones were propagated on selective medium and yeast genomic DNA was isolated by phenol/chloroform purification (as described in [26]) with subsequent precipitation. Alternatively, yeast plasmid DNA was directly purified using the Zymoprep II™ Yeast Plasmid Miniprep Kit (Zymo Research) or the QIAprep Spin Miniprep Kit (Qiagen).

### Southern Blot analysis

Genomic DNA of yeast TAR clones was digested with NotI and separated on 1% agarose gels in 0.5 x TBE using a CHEF mapper system (BIORAD) with the following conditions: forward: 9 V/cm, 10 s initial switch, 40 s final switch, reverse: 6 V/cm, 10 s initial switch, 40 s final switch, for 24 hrs at 14°C.

Southern blot, hybridization and detection were performed as per manufacturer's instruction for non-radioactive nucleic labeling and detection (Roche Diagnostics) using Blocking reagent (Roche), CDP-Star (PerkinElmer or Roche), Maleic Acid (Sigma). The probe consisted of fluorescein-labeled vector backbone (pGEM7, Promega) using Fluorescein-High Prime labeling kit (Roche) and was immunologically detected using Anti-fluorescein-AP, Fab fragments (Roche) on positively charged nylon membranes (Roche).

### Transformation of positive yeast clones into *E. coli* and Restriction Fragment Length Polymorphism (RFLP) analysis

Genomic DNA of individual yeast clones was prepared as described above and transformed into *E. coli* PMC103 either by electroporation or by chemical transformation into ultra competent DH5α cells. Plasmid DNA from bacterial cells was purified using the QIAprep Spin Miniprep Kit (Qiagen).

For RFLP analyses, purified plasmid DNA, of individual clones, were digested with NotI and NdeI. The digests were separated on a 0.7% agarose gel and visualized by ethidiumbromide staining. The vector backbone contains one restriction site each for NotI and NdeI and the number of NdeI-restriction sites varies for different *var* genes, thereby creating a distinct band for the vector backbone and a unique pattern of bands for individual *var* genes. To determine the insert quality, we sequenced the 5′ and 3′ ends of each isolated TAR clone using plasmid DNA (Table 2). In addition, the PCR products obtained from the DBL1alpha colony screen of yeast clones were sequenced. All sequencing was conducted at Macrogen Inc., Korea.

### Next-generation sequencing of three TAR clones

TAR clones upsAIII_10, upsBI_29 and upsCI_3 were selected for tagged deep sequencing. After transformation in *E. coli*, high plasmid concentrations were prepared using the PowerPrep™ HP Plasmid Maxiprep (Marligen Biosciences). Initial sequence analyses were performed by Fasteris SA, Switzerland. Briefly, individual clones were sheared by Fragmentase treatment (NEBNext™ dsDNA Fragmentase™, New England Biolabs) according to the manufacturer's instructions, purified via Qiagen columns and fragments of each clone were labeled with distinct bar-coded adapters to distinguish the reads of the three different TAR clones. High-throughput sequencing was performed on Genome Analyzer GAII, in 1 PE channel; obtaining 2 x 38 bp reads. Bioinformatic analysis included *de novo* assembly, assembly overlap and validation using the following software: VELVET, version 0.7.54, SeqMan, version 8 and MAQ, version 0.7.1.

The obtained sequences of clones upsAIII_10, upsBI_29 and upsCI_3 have been deposited with NCBI with the following Accession numbers HM358637-39.

### Sequence analysis

All alignments were performed with the ClustalW multiple alignment tool (www.ebi.ac.uk/Tools/clustalw2) at default parameters. BLAST analyses were performed against the NCBI nucleotide database or against the non-redundant protein sequences database at NCBI. A The open reading frame of three complete sequences were analyzed using the translation tool of the ExPASy proteomics server (www.expasy.ch) from the Swiss Institute of Bioinformatics (SIB) [41] and the InterProScan Sequence Search of EBML-EBI.

## Supporting Information

Figure S1 Gap closure of clone upsCI_3. (A) Diagram of the structure of the clone with gap and PCR primers designed shown. (B) PCR product covering the gap. (C) Sequence analysis of the PCR product showing AT richness.
(TIF)

Table S1 Distribution of *var* genes within the three *var* groups upsA, upsB and upsC obtained by the TAR experiment compared with *P. falciparum* strains IT4/25/4, 3d7 and HB3 [19], [30]. * For the 3d7 strain, the number of upsB *var* genes include B/A and B/C *var* groups [19].
(DOCX)
