# Supplementary figures and images for: Cloning of the Repertoire of Individual Plasmodium falciparum var Genes Using Transformation Associated Recombination (TAR)
Source: PLoS One. 2011 Mar 7;6(3):e17782. doi: 10.1371/journal.pone.0017782 (PMC3049791; doi:10.1371/journal.pone.0017782)

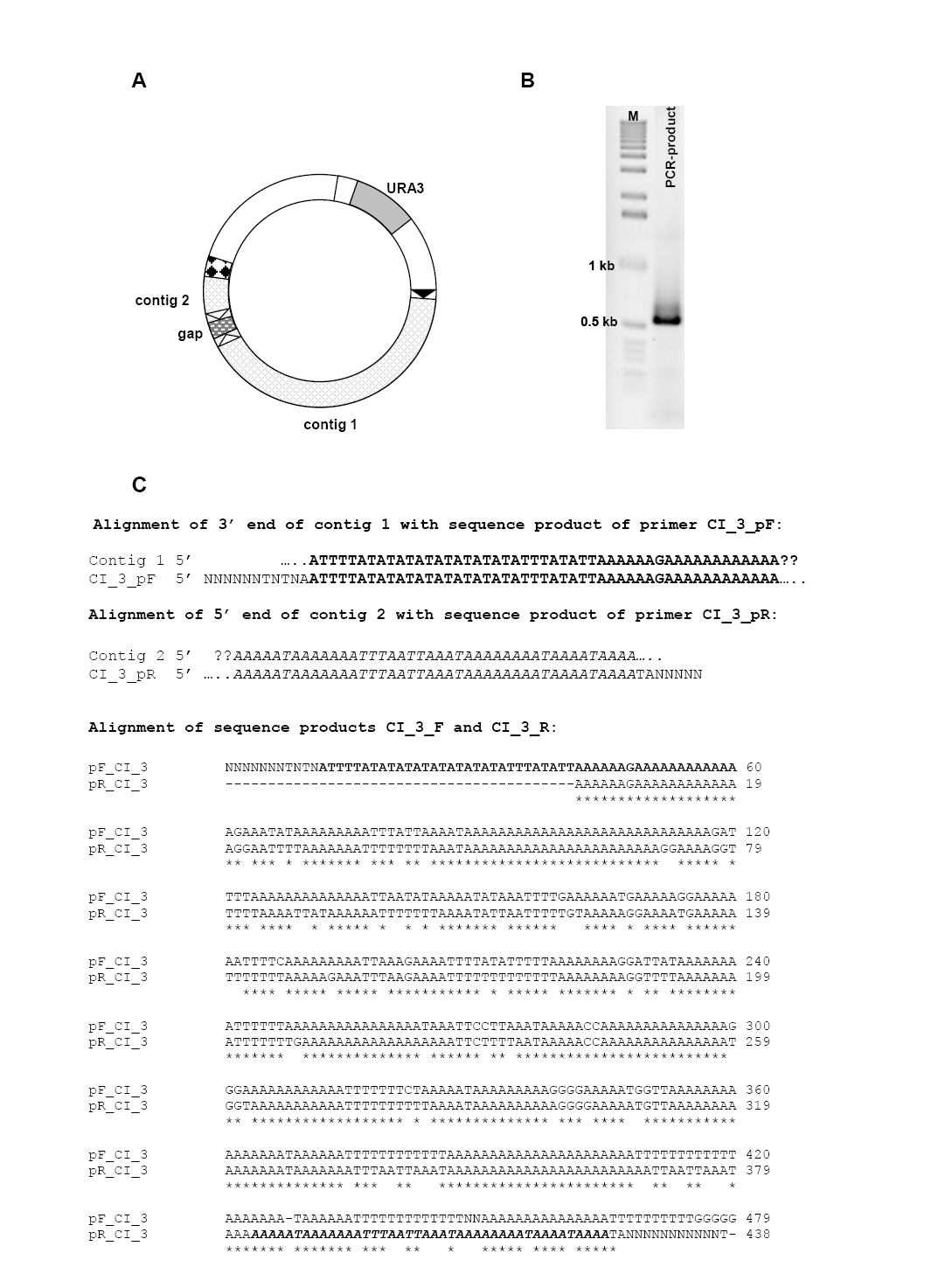

Supplement: Figure S1 — Gap closure of clone upsCI_3. (A) Diagram of the structure of the clone with gap and PCR primers designed shown. (B) PCR product covering the gap. (C) Sequence analysis of the PCR product showing AT richness. (TIF) [file pone.0017782.s001.tif]
