# Supplementary material for: Cloning of the Repertoire of Individual Plasmodium falciparum var Genes Using Transformation Associated Recombination (TAR)
Source: PLoS One. 2011 Mar 7;6(3):e17782. doi: 10.1371/journal.pone.0017782 (PMC3049791; doi:10.1371/journal.pone.0017782)
Supplement: Table S1 — Distribution of var genes within the three var groups upsA, upsB and upsC obtained by the TAR experiment compared with P. falciparum strains IT4/25/4, 3d7 and HB3 [19], [30]. * For the 3d7 strain, the number of upsB var genes include B/A and B/C var groups [19]. (DOCX) [file pone.0017782.s002.docx]

**Table S1**: Distribution of *var* genes within the three *var* groups upsA, upsB and upsC obtained by the TAR experiment compared with *P. falciparum* strains IT4/25/4, 3d7 and HB3 [19,30]. * For the 3d7 strain, the number of upsB *var* genes include B/A and B/C *var* groups [19].

|  | **TAR clones** | **IT4/25/4** | **3d7** | **HB3** |
| --- | --- | --- | --- | --- |
| no of upsA genes | 46 | 10 | 10 | 8 |
| no of upsB genes | 91 | 22 | 35 * | 27 |
| no of upsC genes | 68 | 5 | 13 | 10 |
|  |  |  |  |  |
| **Proportions**: |  |  |  |  |
| upsA genes | 1 | 1 | 1 | 1 |
| upsB genes | 2.0 | 2.2 | 3.5 | 3.4 |
| upsC genes | 1.5 | 0.5 | 1.3 | 1.3 |

The distribution of clones is not significantly different from the distributions of *var* gene classes in sequenced genomes (Chi-squared (6 degrees of freedom) = 0.078, Probability is nearly 1.0).
